# Uncrewed aircraft system spherical photography for the vertical characterization of canopy structural traits

**DOI:** 10.1111/nph.17998

**Published:** 2022-02-22

**Authors:** Vicent Agustí Ribas Costa, Maxime Durand, T. Matthew Robson, Albert Porcar‐Castell, Ilkka Korpela, Jon Atherton

**Affiliations:** ^1^ Optics of Photosynthesis Laboratory Institute for Atmospheric and Earth System Research (INAR)/Department of Forest Sciences Viikki Plant Science Centre (ViPS) Faculty of Agriculture and Forestry University of Helsinki Helsinki 00014 Finland; ^2^ Organismal and Evolutionary Biology (OEB) Viikki Plant Science Centre (ViPS) Faculty of Biological and Environmental Sciences University of Helsinki Helsinki 00014 Finland; ^3^ Department of Forest Sciences Faculty of Agriculture and Forestry University of Helsinki Helsinki 00014 Finland

**Keywords:** plant area density (PAD), plant area index (PAI), spherical photography, structural traits, uncrewed aircraft system (UAS)

## Abstract

- The plant area index (PAI) is a structural trait that succinctly parametrizes the foliage distribution of a canopy and is usually estimated using indirect optical techniques such as digital hemispherical photography. Critically, on‐the‐ground photographic measurements forgo the vertical variation of canopy structure which regulates the local light environment. Hence new approaches are sought for vertical sampling of traits.
- We present an uncrewed aircraft system (UAS) spherical photographic method to obtain structural traits throughout the depth of tree canopies. Our method explained 89% of the variation in PAI when compared with ground‐based hemispherical photography.
- When comparing UAS vertical trait profiles with airborne laser scanning data, we found highest agreement in an open birch (*Betula pendula*/*pubescens*) canopy. Minor disagreement was found in dense spruce (*Picea abies*) stands, especially in the lower canopy.
- Our new method enables easy estimation of the vertical dimension of canopy structural traits in previously inaccessible spaces. The method is affordable and safe and therefore readily usable by plant scientists.

## Introduction

The vertical density of foliage relates to photosynthetic productivity by determining the distribution and interception of sunlight through a plant canopy. As a measure of vertically integrated foliage density, the leaf area index (LAI) is therefore an important canopy structural trait and has been used to model a diverse range of processes including photosynthesis (Duchemin *et al*., 2006) evaporation and transpiration (Jongschaap, 2006; Cleugh *et al*., 2007), and rainfall interception (Dietz *et al*., 2006).

Destructive measurement of LAI, generally defined as the one‐sided leaf area per unit area of ground (Zheng & Moskal, 2009), is laborious and impractical. Indirect optical techniques including hemispherical photography are usually used instead (Jonckheere *et al*., 2004; Majasalmi, 2015). Indirect approaches typically invert the following model to arrive at the LAI:

(Eqn 1) $$P\left(\theta \right)=\exp\left(\frac{‐G(\theta )\cdot \Omega (\theta )\cdot \text{LAI}}{\cos\;\theta }\right)$$

where P(θ) is the probability of a ray of light passing through the canopy without encountering foliage or other plant elements at zenith angle *θ*, and *G* parameterizes the projection of leaf area relative to the zenith direction. It is the gap fraction P(θ) that is measured by the optical instrument (Danson *et al*., 2007). Clumping is accounted for with the clumping index Ω (Nilson, 1971), but Eqn 1 does not explicitly correct for woody elements. In uncorrected form, the LAI is referred to as the plant area index (PAI), which we use here (Chen *et al*., 1991).

In digital hemispherical photography (DHP), a single lens reflex camera is combined with a wide‐angle lens, and possibly levelling equipment to ensure that the camera points directly upwards or downwards (Yan *et al*., 2019). Specialist software is used to segment captured imagery into gap fraction or vegetation, and to derive structural traits via Eqn 1. Importantly, DHP has several limitations that if unaccounted for can produce significant errors. Issues include the requirement for uniform, and usually overcast sky conditions (Leblanc & Chen, 2001); sensitivity to camera settings and hardware, including exposure (Macfarlane *et al*., 2000), camera and lens types (Wagner, 1998), and image format and size (Frazer *et al*., 2001); and choice of postprocessing steps, including gamma correction (Macfarlane *et al*., 2007) and segmentation algorithm (Nobis & Hunziker, 2005). Recent work has shown how the use of raw format image files can overcome some of these issues (Macfarlane *et al*., 2014).

Despite the above limitations, DHP remains a popular option when field estimates of LAI are required (Chianucci, 2020). However, it is an additional limitation that motivated the development of the method described here: the restriction of sampling to on‐the‐ground photography. This limits DHP to places accessible on foot, and more importantly precludes measurement of vertical heterogeneity in canopy structure, a topic of much interest to plant scientists (Disney, 2019). Note that it is the vertical plant area density (PAD), or one‐sided leaf area per unit volume (Hosoi & Omasa, 2006), that dictates the local light environment (Smith *et al*., 2019) and transfer of energy within a canopy (Lalic & Mihailovic, 2004), and this is a trait that is unmeasurable from ground‐based DHP.

For DHP to circumvent the on‐the‐ground restriction requires impractical structures such as ropes (Fauset *et al*., 2017), towers (Leuchner *et al*., 2011; Dengel *et al*., 2015), cranes (Parker *et al*., 2001), portable hydraulic hoists (Canham *et al*., 1994) or balloons (Meir *et al*., 2000; Parker *et al*., 2001), all of which can interfere with measurements. An alternative approach is laser scanning technology, conducted either airborne (ALS; Korhonen *et al*., 2011; Korpela *et al*., 2014) or from terrestrial platforms (TLS; Calders *et al*., 2020). TLS (Hosoi & Omasa, 2006) or ALS (Lim *et al*., 2003; Lovell *et al*., 2003) can be used to retrieve LAI and to model canopy light interception (S. Tian *et al*., 2021) in three dimensions. However, the cost and complexity of laser scanning represent two major drawbacks that prevent its widespread adoption. For plant scientists, laser scanning systems have a high ‘barrier to entry’ (Calders *et al*., 2020).

Recently, uncrewed aircraft systems (UASs) have gained traction as platforms capable of measuring spatial variation in canopy structure (McNeil *et al*., 2016; Brüllhardt *et al*., 2020; Krisanski *et al*., 2020; Umarhadi & Danoedoro, 2021). Laser scanning instrumentation can be mounted on UAS platforms to expand the horizons of ALS from piloted aircraft to smaller, more manoeuvrable systems (Wallace *et al*., 2012; Brede *et al*., 2019; Yin & Wang, 2019). A recent study demonstrated that within‐canopy flight is possible with a laser scanning UAS, although the UAS was relatively large and therefore potentially challenging and risky to fly in dense forest canopies (Hyyppä *et al*., 2021). Additionally, recent studies have shown that above‐canopy ALS‐like canopy trait estimation algorithms can be applied to Structure from Motion photogrammetry data collected from UAS platforms (Brüllhardt *et al*., 2020; Lin *et al*., 2021).

UAS‐based DHP is also in active development, downwards looking in agricultural crop fields (Brown *et al*., 2020) and upwards looking in forest canopies (Brüllhardt *et al*., 2020). The motivation for such systems is the low cost and complexity relative to laser scanning systems, coupled with the access that an airborne system can provide. However, and as with laser scanning, current UAS–DHP approaches require nonintegrated imaging systems and/or relatively large and heavy platforms. We developed the UAS‐based spherical photography method presented here to address these limitations. The new method uses computational spherical photography and relies on standard cameras which are integrated into mass‐produced UAS airframes.

A panoramic spherical image is produced by combining multiple individual images to cover an extremely large angle of view, up to and including the full viewing sphere. To produce a panorama, individual images are algorithmically stitched together and mapped onto a virtual spherical surface, which is subsequently reprojected onto the 2D plane typically using the equirectangular projection (Zhang & Huang, 2021). Spherical panoramas are acquired either with a combination of two wide‐angle lenses via specially designed cameras systems, or by rotating a conventional camera about its horizontal and vertical axes whilst acquiring imagery (Fangi & Nardinocchi, 2013; Barbero‐García *et al*., 2018). In terms of plant science applications, spherical imagery has been used to estimate individual tree heights, diameters at breast height, basal area and canopy openness (Wang *et al*., 2021). Spherical panoramas captured by a mobile phone have also been used to reproduce gap fraction and LAI estimates from a traditional DHP system (Andis‐Arietta, 2021). In these studies, the spherical panoramas were reprojected to hemispherical fisheye projections, which are equivalent to imagery collected by DHP systems.

**Fig. 1:**
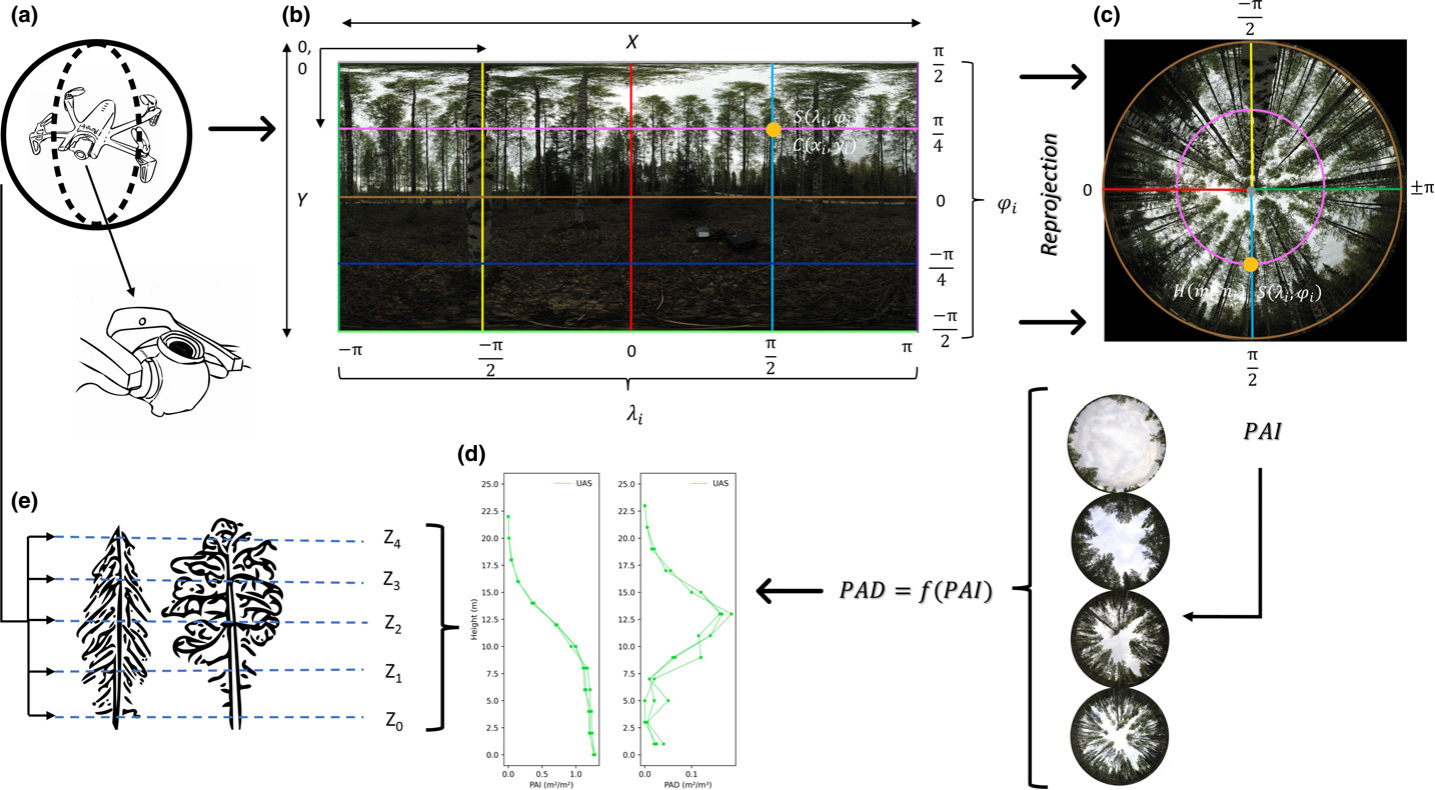
Schematic of the uncrewed aircraft system (UAS) spherical photographic method. The UAS (a) takes spherical panoramas (b) using its gimbal, that are reprojected to hemispherical upward‐looking images (c) using the upper hemisphere (φ > 0). The UAS is flown at different heights of the canopy (e) and therefore a vertical distribution of the trait, e.g. plant area index and density (PAI, PAD), is obtained (d). For details on the reprojection see Supporting Information Notes S1.

In addition to mobile phones, panoramic photography is also possible using mass‐produced UASs. However, most off‐the‐shelf systems cannot be readily used as a full 180° vertical field of view is required, a feature that is relatively rare as it requires a specialized gimbal capable of rotating the camera viewing direction upwards (see Fig. 1a inset drawing of a UAS sensor). Our main goal was to replace cumbersome and restrictive DHP equipment with miniaturized UAS technology capable of imaging the full 180° vertical field of view and therefore retrieving vertical information related to canopy structural traits, driving light and functional gradients within a plant canopy environment. To validate our methodology, UAS‐mounted spherical imagery was compared with traditional DHP across species and stand types. To improve on DHP, vertical profiles of PAD were derived, which were compared to airborne laser scanning data. Fig. 1 provides an overview of the new method.

## Materials and Methods

### Overview of measurements

There were two main objectives to the data collection:

1. comparison of UAS‐estimated PAI and gap fraction with DHP;
2. retrieval of vertical trait profiles using UAS.

To achieve these objectives, measurements were conducted at two sites in Finland during spring and early summer 2021. The UAS was flown through mature Norway spruce (*Picea abies* (L.) H. Karst), Scots pine (*Pinus sylvestris* L.) and silver/downy birch‐dominated (*Betula pendula* Roth/*Betula pubescens* Ehrh) stands, taking spherical images at different heights. Vertical profiles of PAI were used to derive PAD and compared to ALS data, and DHP was used to validate near‐ground imagery. Data were collected at the Viikki Arboretum (Helsinki – 60.2°N, 25.0°E) of the University of Helsinki between February and April 2021, and in the surroundings of the Station for Measuring Ecosystem‐Atmosphere Relations II (SMEAR II), Hyytiälä (61.8°N, 24.3°E) in May and June 2021. Spherical panoramic imagery was reprojected using a Python script and analysed using Hemisfer software v.3.1 (Patrick Schleppi, WLS Swiss Federal Institute for Forest, Snow and Landscape research, Switzerland) to optically derive the gap fraction and estimate PAI. The same software was used for the DHP data.

### UAS description and camera calibration

The mass‐produced Parrot ANAFI drone was used to capture spherical panoramas (Parrot Drones SAS, Paris, France). The ANAFI is compact (240 mm maximum dimension) and lightweight (320 g), with a high‐resolution camera (21 million pixels) capable of looking directly upwards thanks to its 180° tilt gimbal (Fig. 1). The main characteristics of the UAS are given in Table 1.

**Table 1:** Summary of uncrewed aircraft system (UAS) characteristics.

UAS characteristics	
*Physical properties*	
Size unfolded	175 × 240 × 65 mm
Weight (take‐off weight)	320 g
Maximum flight time (one battery)	25 min
Operating temperature range	−10 to 40°C
Satellite positioning systems	GPS and GLONASS
*Imaging system*	
Sensor	1/2.4″ CMOS: 5.9 × 4.43 mm
Lens	ASPH (sharper images)
Aperture	f/2.4
Focal length (specifications)	Photo: 3.92–11.76 mm
Shutter speed	electronic shutter 1–1/10 000 s
ISO range	100–3200
Image resolution	
Wide	21 megapixels (5344 × 4016)/4 : 3/84° HFOV
Rectilinear	16 megapixels (4608 × 3456)/4 : 3/75.5° HFOV.IHFOV: 0.016°–IVFOV: 0.022°
Image formats	JPEG, DNG (raw)
*Stabilization*	
Image stabilization	3‐axis hybrid: Mechanical – 2‐axis Roll/Tilt angles; Electronic (EIS) – 3‐axis Roll/Pan/Tilt angles
Gimbal tilt	Controllable –90° to +90° (180° total)
*Additional information*	
Cost in 2021 (Finland)	650–800 Euros
Pending EULA class	C1

To calibrate the ANAFI camera, we followed the protocol found in the hemisfer documentation (Schleppi *et al*., 2007; Thimonier *et al*., 2010). The output of the calibration is a lens function which characterizes radial distortion in the reprojected hemispherical image (see Supporting Information Notes S2; Fig. S1). This was performed outside under a small bridge, as the ANAFI stitching method failed indoors due to movement of the UAS. Minimal radial distortion was found in the UAS sensor, and hence we proceeded with the built‐in linear lens function to analyse UAS imagery.

### UAS imagery acquisition and processing

Each UAS‐based spherical image was formed from 42 images in JPEG format taken automatically using the 360° image mode. These were stitched together using either the inbuilt Panorama mode in the FreeFlight 6 Parrot flight application (Parrot Drones SAS) or Microsoft Image Composition Editor (Microsoft Corp., Redmont, WA, USA). The latter was used when FreeFlight 6 failed due to software processing errors. The resulting spherical images were in the equirectangular projection, where the *x*‐axis represents the azimuth angle and the *y*‐axis the zenith angle (Fangi & Nardinocchi, 2013). The built‐in UAS sensors (gyroscope, compass and barometer) allow the spherical image to be accurately levelled, and thus the upper and lower half correspond to the upper and lower hemisphere (Li & Ratti, 2019). A Python script (https://github.com/HowcanoeWang/Spherical2TreeAttributes, Wang, 2019) was adapted to reproject panoramas to DHP equivalent imagery (see Notes S1). These UAS‐based hemispherical images had a size of 4000 × 4000 pixels and a radius of 2000 pixels.

To process UAS imagery, we used the hemisfer built‐in linear lens function with a 90° field of view. The Nobis & Hunziker (2005) method was used to binarize the image into black (plant material) and white (sky) pixels. It was binarized either manually or following the Ridler & Calvard (1978) method when the former failed in open and heterogenous sky conditions. The gamma value was set by default at 2.2 and all bands of the RGB image were used. The gap fraction was calculated by dividing the hemispherical image into five annuli of 15° and the Miller (1967) method was used for PAI. Shoot‐level clumping was corrected by dividing the PAI by four times the mean Silhouette to Total Area Ratio (STAR) (Oker‐Blom & Smolander, 1988). A star value of 0.147 was used for pine stands and 0.161 for spruce. Clumping correction was not applied to birch (Majasalmi *et al*., 2013) and we did not correct for stand‐level clumping. We also did not correct the woody‐to‐nonwoody ratio, as the purpose was not to achieve accuracy in LAI estimation but rather to assess the relative accuracy of the UAS method (Brüllhardt *et al*., 2020).

For the UAS–DHP intercomparison, UAS imagery was taken at the same height as DHP, as close in time as possible. After taking off and stabilizing the drone, the spherical image protocol was started. Exposure settings were set to optimize the final image: ISO 200 and light‐dependent shutter speed (set to optimize first image), manually set looking at the UAS histogram. After capture, images were inspected manually and then run through hemisfer to produce estimates of gap fraction and PAI.

### DHP data collection for comparison with UAS imagery

We collected DHP imagery coincident with UAS‐based spherical images and taken under differing plant canopy architectures at both sites (total *n* = 60). DHP images were taken with a single lens reflex camera, an extreme‐wide field of view fisheye lens, a self‐levelling mount with circular bubble level and a tripod. The camera was a Canon EOS 70D (Canon Inc., Ōta, Tokyo, Japan). The fisheye lens was a Sigma 4.5 mm F2.8 EX DC Circular Fisheye HSM (Sigma Corp., Kawasaki, Kanawanga, Japan), with a full 180° field of view, a focal length of 17–55 mm and minimum aperture of F22. The self‐levelling mount was a Delta‐T SLM9 (Delta‐T Devices Ltd, Cambridge, UK) which was mounted on top of a Slik Pro 400DX tripod (Slik Corp., Hidaka, Hokkaido, Japan).

The camera was mounted at 100 cm height from the ground, aligned towards magnetic north and levelled. Exposure was optimized using the image histograms (Beckschäfer *et al*., 2013), exposing the image to the brightest pixels. ISO was set to a constant value of 200 to avoid grain, the sensor openness was also set to a constant value of F20/22 (Hartikainen *et al*., 2018), and the focus was set to optimize the clarity and quality of the image. As above, hemisfer was used to process imagery. DHP images had a size of 3648 × 3648 pixels and radius of 1530 pixels and a vertical and horizontal Instantaneous Field of View (H/VIFOV) of 0.118°. As with the UAS, the hemisfer calibration protocol was followed, and in this case we used the estimated lens function in our subsequent analysis (Fig. S1).

### PAD estimation

To obtain vertical profiles of PAI and PAD, we first selected suitable locations for flying vertical profiles requiring canopy gaps of at least *c*. 12 m^2^. Three repetitions of two profiles per main species were conducted by taking three images at each height, starting at the minimum flight height (< 50 cm) and then flying upwards and stopping at 2 m increments. PAI profiles were calculated as above using images taken at each measurement height. We derived PAD profiles from adjacent‐in‐height PAI values using the following formula (Neumann *et al*., 1989):

(Eqn 2) $$\text{PAD}\approx \frac{{\text{PAI}}_{i}‐{\text{PAI}}_{j}}{z_{j}‐z_{i}}$$

where *i* is the first layer (lower height), *j* is the second layer (upper height) and *z* is height from the ground.

### Comparison of vertical PAD profiles to ALS data

We used ALS data to assess the accuracy of our PAI and PAD profiles. ALS acquisition took place on 3 June 2020 using an aeroplane‐mounted Riegl Q1560 sensor (*λ* = 1064 nm). The scanning range was 1.2 km and the area was covered by three overlapping strips. Scan zenith angles were 1–24° and the pulse density was 40–60 pulses m^–2^. The sensor operates two laser scanners. These had been calibrated to provide a 3D relative point match (*c*. 68% precision) of better than 20 cm. This was verified against man‐made targets such as power line cables. Each pulse transmitted resulted in one to nine echoes (discrete points) with a minimal spacing (along the pulse) of *c*. 1.5 m.

To calculate a PAD proxy from LiDAR point cloud data, we first excluded points greater than 10 m radius from the UAS profile location. Points below 1.5 m height were assigned as ground returns $\left(N_{g}\right)$. Next, the cylindrical point cloud was divided into 50 vertical segments, which corresponded to an *c*. 0.5 m height interval at each location. We used the ratio of points above each level to total returns, which included ground returns, from Solberg *et al*. (2006) to estimate transmission (*T*) through the canopy:

(Eqn 3) $$T=1‐\frac{N_{c}}{N_{t}}=1‐\frac{N_{c}}{N_{g}+N_{c}}$$

where *N*_c_ is the returns above a certain height and *N*_t_ is the total number of returns. The following equation was then used to estimate ALS PAI:

(Eqn 4) $$\text{PAI}=\frac{‐1}{k}\cdot \log_{e}\left(T\right)$$

where *k* is the extinction coefficient value. (Note that Eqn 4 is a modified form of Eqn 1 assuming *T* is equal to *P*.) We estimated *k* and PAI from Eqn 4 using ordinary least squares and UAS PAI measurements. The differential values of PAI throughout the canopy height were used to estimate the PAD values using Eqn 2, and summary statistics were calculated between ALS and UAS PAD profiles.

### Statistical and error analyses

Linear regression was used to analyse the relationship between DHP and UAS estimated traits. The coefficient of determination (*R* ^2^) and the regression standard error were calculated. The relative regression standard error (RSE) was calculated as:

(Eqn 5) $$\text{RSE}=\frac{\text{SE}}{\bar{y}}\cdot 100=\frac{\sqrt{\frac{1}{N‐2}\cdot \sum_{i=1}^{N}{\left(y_{i}‐{\hat{y}}_{i}\right)}^{2}}}{\bar{y}}\cdot 100$$

where $\bar{y}$ is the mean value of the DHP trait observations, *y_i_* is the observed DHP values, ${\hat{y}}_{i}$ is each of the predicted UAS values and *N* is the number of observations, with the traits being either gap fraction or PAI.

We also investigated three categories of error in DHP and UAS images: exposure variability, image resolution and stitching error. To investigate exposure errors, the histogram values of each picture were analysed. The difference in mean value of the pixels (using the blue band) for each image was related to the difference in gap fraction between the DHP and UAS images. A relationship between these two values suggests that part of the unexplained variance of the model is attributable to exposure. Linear regression analysis was used the quantify the relationship. Additionally, and for UAS imagery only, we conducted repeated sampling at eight locations. This was needed because for UAS imagery the exposure was set at the start of the data collection and a single set of panoramic imagery takes 2–3 min to collect.

Error could also have resulted from differences in image resolution between the two systems, and hence the computer vision algorithm Structural Similarity Index Measure (SSIM) from the Scikit‐image Python package (Van der Walt *et al*., 2014) was used to highlight differences between DHP and UAS imagery. Finally, we analysed stitching error due to discontinuities in the matching of individual photos in the spherical image‐building process. These errors were defined qualitatively as unnatural vegetation or image discontinuities, and four examples are shown in Fig. S2. To quantify this, all 60 UAS‐based hemispherical images were visually inspected to assess the relative importance of this error. The percentage of photos with no error and more than one error was obtained, as well as the average number of errors per image. To test the significance of the error, we performed a Welch’s *t*‐test (significance level of 0.05) on the residuals of the linear regressions between DHP‐ and UAS‐based imagery of gap fraction and PAI linear models, split by the error rate (no error, or more than one error).

## Results

### Comparison of reprojected spherical imagery with on‐the‐ground hemispherical imagery

**Fig. 2:**
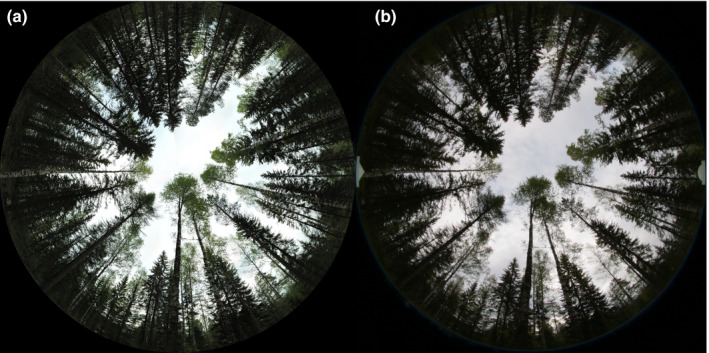
Example comparison of hemispherical imagery using the two differing approaches: (a) is an image from the uncrewed aircraft system (UAS) and (b) is the digital hemispherical photography (DHP) image. Images were taken in May 2021 close to Hyytiälä SMEAR II station, in a thinned stand of Scots pine, birch and Norway spruce.

Fig. 2 is a comparison of imagery taken using the two systems under a mixed birch, pine and spruce canopy. Qualitatively, the UAS imagery is similar in composition to the DHP system, yet there are also subtle differences such as the higher resolution of the UAS image.

**Fig. 3:**
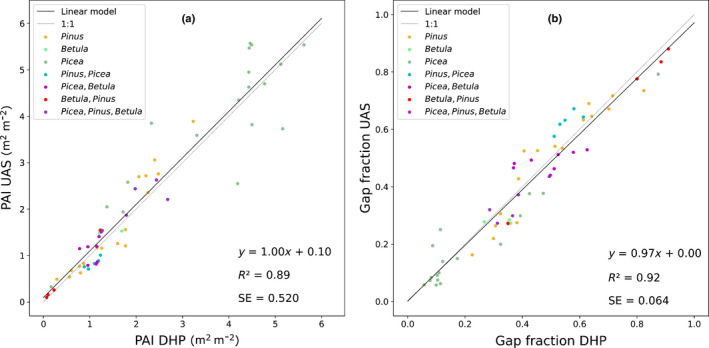
Comparison between the uncrewed aircraft system (UAS)‐ and digital hemispherical photography (DHP)‐derived plant area index (PAI) (a) and gap fraction (b). A total of 60 locations (*n* = 60) were selected, of which 22 pairs of images were in Viikki Arboretum. The following canopy structures were represented: open field, unthinned dense Norway spruce, mixed Scots pine and birch, pure birch, mature Scots pine, Norway spruce and birch, and thinned pure Scots pine.

The main results of the comparison between the UAS‐based method and the DHP datasets are shown in Fig. 3. The coefficient of determination between the two methods was 0.89 for PAI and 0.92 for gap fraction across all annuli. The SE of the UAS‐based PAI was 0.520 m^2^ m^−2^, which in percentage terms gives an RSE of 24.2%; in terms of gap fraction estimation, the RSE was 16.1%.

### Error analysis of gap fraction

**Fig. 4:**
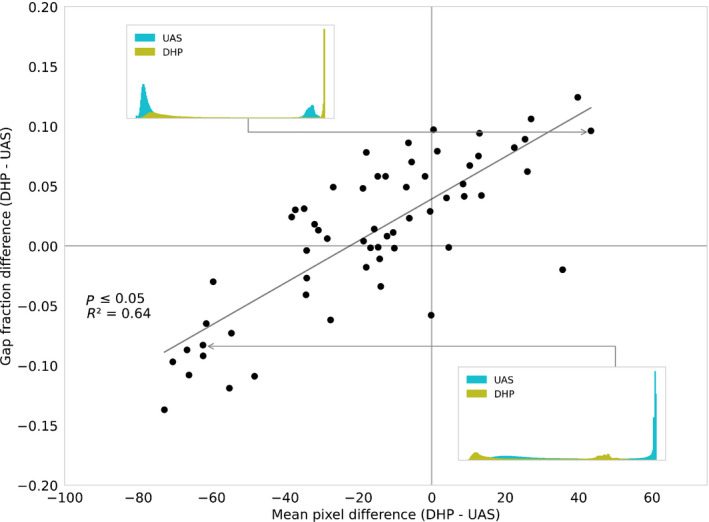
Relationship between gap fraction difference and the histogram mean pixel value difference between the digital hemispherical photographic (DHP) imagery and the uncrewed aircraft system (UAS) imagery for the blue channel. The inset plots are examples of blue channel image histograms, with values from 0 to 255, demonstrating overexposure in each of the two systems.

We conducted an error analysis to pinpoint the cause of differences in DHP and UAS gap fraction and PAI estimates. Starting with exposure, we found a coefficient of determination of *R* ^2^ = 0.64 (Fig. 4) between mean pixel values and gap fraction differences between DHP and UAS, suggesting a probable influence of exposure on the results. In Fig. 4, negative values indicate that DHP was underexposed relative to UAS and positive values indicate that DHP was overexposed relative to UAS.

Next, we analysed the replicability of gap fraction estimates in UAS imagery. The results showed that the variability between repetitions remained approximately constant at each location, with a median deviation of 3.4% for all locations (median of maximum deviation between the three measurements divided by its average, per location). Most repetitions (87.5%) demonstrated a deviation in gap fraction of < 10%.

**Fig. 5:**
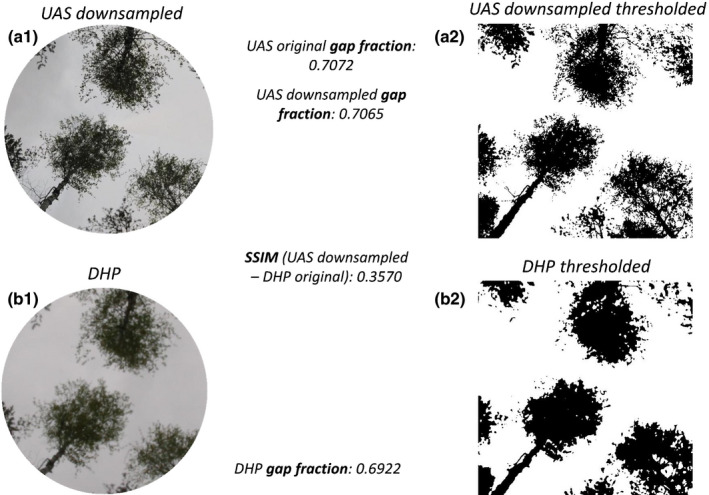
Detail of the comparison of the first annulus of two images (zenith angle between 0° and 15°). Top row, uncrewed aircraft system (UAS) downsampled image (a); bottom row, digital hemispherical photography (DHP) image (b). From left to right: direct subimage comparison (a1, b1) and comparison of the binarized image (a2, b2). The gap fraction for each subimage was obtained, as well as the Structural Similarity Index Measure (SSIM), in order to quantitatively compare the two images. The UAS image was downsampled to the same number of pixels as the DHP image.

The next source of error studied was the image resolution and resulting quality difference. The increased resolution of the UAS was evident when comparing images side by side (Fig. 2) and has been previously noted in reprojected panoramic imagery by Andis‐Arietta (2021). Fig. 5 provides a closer view of a small section of the example comparison imagery that highlights this issue and shows structural image differences, which are summarized by the SSIM value. Although the UAS‐based hemispherical image had an IFOV of 0.09°, whereas DHP had a value of 0.118°, in the analysis in Fig. 5 the UAS image was downsampled to the same number of pixels (IFOV) as the DHP. The quality difference persisted even when the number of pixels were set as equal.

The final source of error studied was stitching error. We found that 50% of images were visually clear of errors, and the average number of errors per image was 1.07. The results of the Welch’s *t*‐test performed on the PAI and gap fraction residuals, grouped into errors present and no errors are shown in Table 2. There were no statistically significant differences between the groups for PAI or gap fraction.

**Table 2:** Results of the statistical tests relating to panoramic stitching errors in plant area index (PAI) and gap fraction estimates.

Statistics	Residuals PAI		Residuals gap fraction	
	No error	Error	No error	Error
Mean	0.0038	−0.0023	0.0025	−0.0025
Variance	0.2842	0.2597	0.0042	0.0040
*P* value (Welch’s test, two‐tailed)	0.964		0.765	

### Vertical profiles of PAI and PAD

**Fig. 6:**
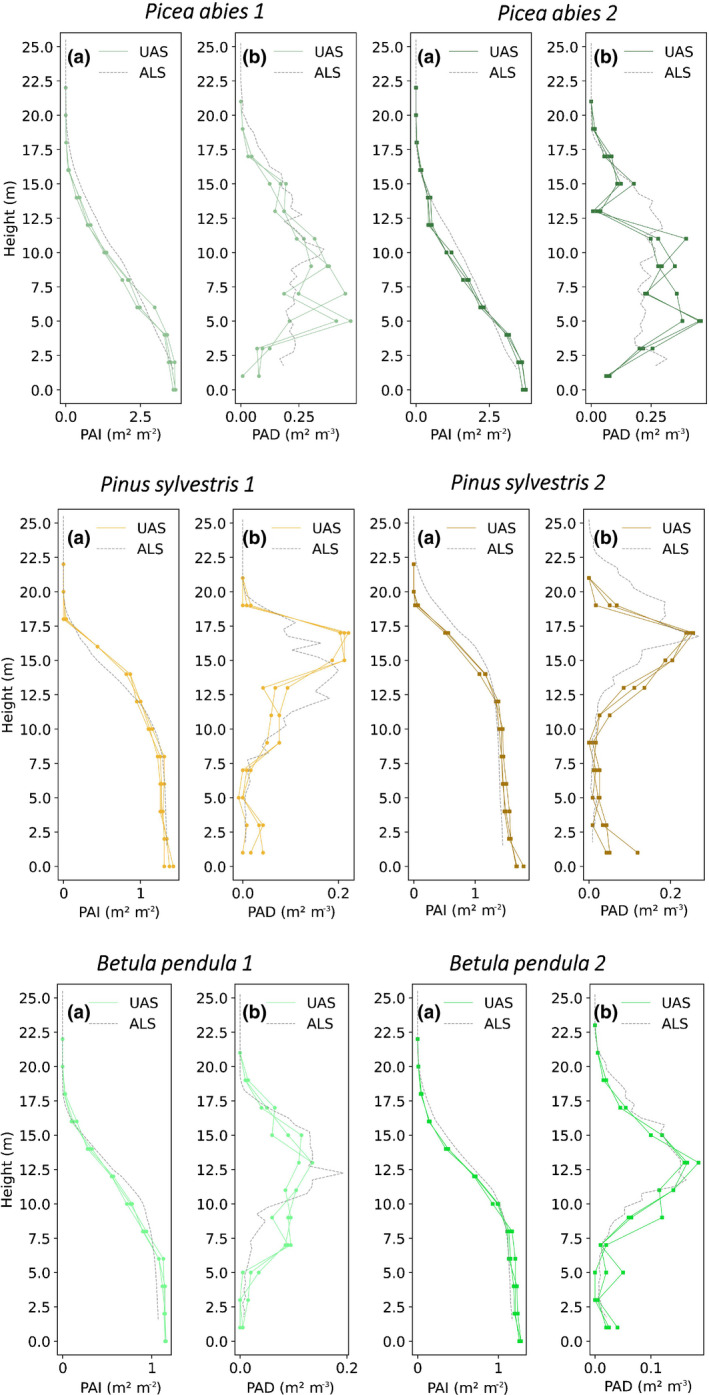
Vertical profiles obtained from uncrewed aircraft system (UAS) spherical imagery (continuous lines) and airborne laser scanning (ALS) data (grey dashed lines). Each row shows two different profiles in a *Picea abies* stand, *Pinus sylvestris* stand and *Betula pendula*‐dominated stand. Letters represent the cumulative plant area index (PAI) (a) and plant area density (PAD) (b) vertical distribution. In each profile location, three repetitions (spherical photos) were taken per layer (height).

Next, we used the UAS method to retrieve vertical profiles of PAI and PAD in different canopies. The results are presented in Fig. 6, which shows six vertical profiles, two per species, obtained in a Norway spruce stand, a Scots pine stand and a birch‐dominated stand. The figure also shows the ALS proxies of PAI and PAD.

UAS PAD profiles in the top row of Fig. 6 demonstrate the characteristic conical shape of a dense Norway spruce stand. By contrast, in *P. sylvestris* 1 and 2, the typical lollipop‐like elliptical profile of Scots pine is evident in the PAD estimate. The stand was thinned in 2020, resulting in an opened stand, with a low PAI value and trees with few branches below the height of the crown. Finally, the *B. pendula* shown on the bottom row of Fig. 6 differs in crown shape in comparison with the other two species. The birch trees had longer crowns compared to the pines.

ALS PAI and PAD proxy profiles are shown as grey lines in Fig. 6 and estimated ALS extinction coefficients (*k*) and statistical summary results between the two methodologies are presented in Table 3. Estimated extinction coefficients were lowest in spruce (0.30, 0.35) and highest in birch (0.45, 0.61). Comparisons between ALS and UAS showed similar PAD profile shapes across species, although there was some divergence at the lower levels of the two spruce sites, which had the largest residual sum of squares between ALS and UAS profiles.

**Table 3:** Statistical analysis results of the ALS–UAS comparison.

Profile	PAD residual sum of squares (m^2^ m^−3^)	Height of maximum PAD residual (m)	*k* estimated
*Picea abies* 1	0.055	5	0.30
*Picea abies* 2	0.116	13	0.35
*Pinus sylvestris* 1	0.025	13	0.37
*Pinus sylvestris* 2	0.022	21	0.48
*Betula pendula* 1	0.008	7	0.45
*Betula pendula* 2	0.003	9	0.61

Note

that in the statistical comparison, ALS plant area density (PAD) values were calculated using the same height step size as the UAS PAD values.

## Discussion

### Evaluation of UAS spherical photography

A major limitation of hemispherical photography is that it is usually restricted to ground‐based imagery, precluding the estimation of vertical traits. This limitation has stimulated recent research into UAS‐based hemispherical image collection (Brown *et al*., 2020; Brüllhardt *et al*., 2020). Such systems use self‐mounted cameras, which are potentially problematic to fly under the forest canopy. By contrast, the low‐cost UAS used here is small, lightweight and has an integrated imaging system that can produce upwards looking imagery. Using our method, hemispherical fisheye‐type images are reproduced by utilizing a computational imaging technique, rather than the bulky DHP hardware used in other studies. This raises the question: does the use of panoramic imagery result in lower quality hemispherical imagery?

The correlations shown in Fig. 3 demonstrate that the spherical panorama‐based estimation of the gap fraction and PAI is very similar to that obtained by ground‐based hemispherical photography across a range of species and canopy structures. Nonetheless, the differences in gap fraction and PAI between UAS and DHP imagery that we did find were related to a number of factors common to both systems.

As expected, we found that exposure differences between systems had a nonnegligible effect on the gap fraction estimation (Fig. 4). Overexposure of imagery results in an overestimation of gap fraction, as small branches or foliage elements are incorrectly segmented as sky. An example of an overexposed DHP histogram is shown in the top left corner inset of Fig. 4. There were also a few overexposed UAS images in the dataset, with an example histogram shown as an inset in the lower right corner of Fig. 4. This inset graph also shows an underexposed DHP histogram. Note that exposure can be difficult to set correctly in panoramic imagery, as the parameters are set at the start of image collection and then remain constant across all camera viewing angles. From our analysis, exposure variability appeared to influence DHP and UAS imagery, but as we arrived at our conclusions by comparing two uncalibrated cameras a note of caution is required.

A further issue related to exposure and UAS measurements was our strong preference for overcast days rather than clear dusks and dawns. This was because complete profiles could take more than 40 min to collect, during which time dusk or dawn light conditions change substantially. Raw format imagery could help with exposure‐ and light condition‐related errors (Macfarlane *et al*., 2014; Hartikainen *et al*., 2018), bypassing automatic gamma correction performed by the camera, but custom software would need to be developed for the UAS to collect raw format panoramas.

The lower quality of DHP imagery relative to the UAS resulted in a reduced number of small gaps. Even when downsampling reprojected panoramic imagery to the same number of pixels as DHP imagery, the angular resolution of the UAS was lower than the DHP, resulting in a higher image quality. In the thresholded binary images, lower quality leads to a greater proportion of misclassified pixels, and hence to an underestimation of small gaps (Macfarlane, 2011; Andis‐Arietta, 2021). This is known as ‘blooming’, occurring when light saturation on the sensor spills over onto neighbouring pixels, and is enhanced by overexposure, which occurred in a few of our DHP images (Leblanc *et al*., 2005). To summarize, the lower resolution of DHP results in a lower gap fraction and accordingly a higher PAI. This pattern was found to exist independently of canopy species and structures.

Stitching errors were found to be of less importance than exposure or resolution. Though generally high, stitching quality depended on the UAS stability when taking the individual images. This was probably related to turbulence in the crown layer (Brüllhardt *et al*., 2020). We therefore encourage careful inspection of imagery before gap fraction estimation for stitching errors, such as those presented in Fig. S2. A final source of error that we did not consider was reprojection error. In theory, the spherical‐to‐hemispherical transformation could cause errors or loss of information, especially in the top of the image where the highest expansion and compression occur.

We used reprojected imagery in our study for two reasons. First, it meant we could compare imagery directly to that captured by a conventional DHP system. The second, and related, reason was that we could use standard software, hemisfer, to compute gap fractions and PAI. Andis‐Arietta (2021) stated the hemispherical reprojection is not a requirement for PAI or gap fraction estimation from panoramas, but did not calculate gap fractions directly from panoramic imagery. Wang (2019) did calculate gap fractions directly from panoramic imagery, using an area‐based weighting function to correct for the cylindrical panoramic projection. Interestingly, and almost three decades ago, Andrieu *et al*. (1994) calculated bidirectional gap fractions from panoramic‐like imagery reprojected from fisheye imagery, effectively inverting the projection applied here. Taken together, these studies suggest a fruitful path forward would be the development of software, and appropriate theory, to operate directly on the common panoramic projections, and relatedly, the fisheye image.

### Vertical profiles of canopy structural traits and ALS comparison

We retrieved vertical profiles of PAI and PAD by flying the UAS in canopy gaps. Although more stable in heavy winds, it is unlikely that larger UASs carrying heavier payloads (Brüllhardt *et al*., 2020; Hyyppä *et al*., 2021) can fly with a similar degree of safety in such areas. However, before we can reach a potential lower limit of gap size with our UAS in the upper canopy, the issue of UAS drift requires attention.

UAS drift was a source of variability in vertical profiles of structural traits. When ascending throughout the canopy, the UAS occasionally drifted from the starting location in the horizontal plane. This was the main reason why large enough gaps were required when choosing the sampling location. Between 6 and 10 m height, drifting was a critical issue, as that height was where the largest crown diameter was situated. Drift is a potential cause of stitching errors, which show up as image discontinuities (Andis‐Arietta, 2021) related to alignment errors (Fangi & Nardinocchi, 2013). A further effect of drifting was to change the sampling location at different heights, potentially altering the PAI value and in extreme cases halting image collection. Further research is needed to better understand the causes of UAS drifting behaviour and to program the UAS so that spherical image collection would not stop even when the pilot manually changed the UAS position.

To explore the effectiveness of our new method, we compared UAS‐based spherical photography‐derived PAD profiles to simple ALS‐estimated PAI and PAD proxies. In general, pine, birch and spruce profiles were well matched between the UAS and ALS approaches. Though dependent on the specific ALS instrumentation and proxy method applied (Korhonen *et al*., 2011), estimated extinction coefficients were similar to literature values, with the birch plots closest to the theoretical spherical leaf angle distribution‐derived *k* of 0.5 (Lintunen *et al*., 2013). This is not surprising as we used UAS imagery to estimate extinction coefficients, but the parameter estimation does demonstrate a novel use of the UAS profiles.

The largest errors between ALS and UAS PAD profiles occurred in the denser spruce plots. Differences between ALS and UAS profiles could relate to a saturation effect at higher LAI values (L. Tian *et al*., 2021) or to differences in the measurement geometries of the two systems, including the effect of laser scan angles. The UAS samples a cone‐like area around the imaging system, whereas the ALS data were restricted to points collected within a 10 m radius cylinder, centred at the UAS location (Fig. S3). Hence, the laser scanned surface was considerably smaller than the surface imaged from the UAS (Korhonen *et al*., 2011). This could have contributed to errors in the method comparison. Using only the inner annuli of the UAS imagery could have potentially lessened the error in the comparison (Solberg *et al*., 2006), but we deemed this step unnecessary based on the strength of our results. Likewise, a more sophisticated PAD method could have been applied (Hosoi & Omasa, 2006; L. Tian *et al*., 2021).

### Expanding the horizons of UAS spherical imagery

The main advance of our method is the ability to capture imagery under a canopy some distance away from the researcher. Aside from the vertical profiles, there are several interesting applications for this. Most obviously, the method can be used to produce hemispherical photographs in previously inaccessible locations. A specific example from our own research is the requirement for hemispherical photography adjacent to light‐exposed upper crowns, where we were previously limited to locations with tower infrastructure. Other examples of difficult to access locations include wetland canopies that are partially flooded with water such as marshes, swamps or mangroves, with the UAS potentially piloted from a boat. Forests with thick understoreys are another example, as are dangerous or pristine environments where human impact should be minimized, keeping in mind that the range of any UAS is potentially restricted by battery capacity, controller signal strength and local legislation.

There are several additional parameters that can be calculated from hemispherical or panoramic photography that we did not investigate here. These include basal area estimation (Wang *et al*., 2021), leaf angle distribution (Qi *et al*., 2019), clumping (Andis‐Arietta, 2021), and the fraction of vegetation cover or intercepted photosynthetically active radiation (Li *et al*., 2015). Both understorey (Law & Waring, 1994) and crop PAI (Brown *et al*., 2020) could be estimated with the lower portion of the spherical panorama. Above‐canopy Structure from Motion photogrammetry, from which it is now possible to estimate LAI profiles (Lin *et al*., 2021), could be complemented with within‐canopy spherical photogrammetry (Fangi & Nardinocchi, 2013). Finally, a recent study showed the potential of upward‐looking thermal imaging to obtain canopy temperatures, which relate to transpiration (Su *et al*., 2020). A UAS thermal approach could be developed, with the caveat that complementary visible imagery should be used for binarization.

### Conclusions

We have demonstrated a new method that used UAS‐based spherical panoramic imagery to estimate vertical profiles of PAI and PAD from canopies of different tree species and structures. The method is inexpensive and safe relative to laser scanning and larger UAS operation. The technique is complementary to laser scanning, which has a higher barrier to entry (Calders *et al*., 2020), and is readily deployable by the working plant scientist.

## Author contributions

VARC wrote the manuscript, performed the analyses and carried out the fieldwork. MD and TMR contributed to the manuscript revision and guided the analyses. AP‐C planned the field sampling with VARC and contributed to conceptualization and manuscript revisions. IK provided ALS data, helped with statistical analyses and contributed to the manuscript. JA conceptualized the study, supervised VARC and helped VARC to write the manuscript.

## Supporting information


**Fig. S1** Calibration functions for both sensors.
**Fig. S2** Four examples of UAS panoramic stitching error.
**Fig. S3** ALS plot point clouds and ground‐level UAS imagery.
**Notes S1** Panoramic reprojection to fisheye imagery.
**Notes S2** Calibration of the imaging sensors.
**Notes S3** Supporting Information references.
Please note: Wiley Blackwell are not responsible for the content or functionality of any Supporting Information supplied by the authors. Any queries (other than missing material) should be directed to the *New Phytologist* Central Office.

## Data Availability

Data and processing scripts associated with the study were deposited on Zenodo under doi: 10.5281/zenodo.5877899.
