# Supplementary material for: Uncrewed aircraft system spherical photography for the vertical characterization of canopy structural traits
Source: New Phytol. 2022 Feb 22;234(2):735–47. doi: 10.1111/nph.17998 (PMC9303749; doi:10.1111/nph.17998)
Supplement: Supplementary file 1 — Fig. S1 Calibration functions for both sensors. Fig. S2 Four examples of UAS panoramic stitching error. Fig. S3 ALS plot point clouds and ground‐level UAS imagery. Notes S1 Panoramic reprojection to fisheye imagery. Notes S2 Calibration of the imaging sensors. Notes S3 Supporting Information references. Please note: Wiley Blackwell are not responsible for the content or functionality of any Supporting Information supplied by the authors. Any queries (other than missing material) should be directed to the New Phytologist Central Office. [file NPH-234-735-s001.pdf]

New Phytologist

***New Phytologist* Supporting Information**

Article title: UAS spherical photography for the characterisation of vertical canopy structural traits

Authors: Vicent Agustí Ribas Costa[1], Maxime Durand[2], T Matthew Robson[2] Albert Porcar-Castell[1], Ilkka Korpela[3], Jon Atherton[1]*
[1]Optics of Photosynthesis Laboratory, Institute for Atmospheric and Earth System Research (INAR)/ Dept. of Forest Sciences, Viikki Plant Science Centre (ViPS), Faculty of Agriculture and Forestry, University of Helsinki, 00014, Finland
[2]Organismal and Evolutionary Biology (OEB), Viikki Plant Science Centre (ViPS), Faculty of Biological and Environmental Sciences, University of Helsinki, 00014, Finland
[3]Dept. of Forest Sciences, Faculty of Agriculture and Forestry, University of Helsinki, 00014, Finland
*Corresponding author, jon.atherton@helsinki.fi

Article acceptance date:
19 December 2021
The following Supporting Information is available for this article:

Notes S1 Panoramic reprojection to fisheye imagery.

Notes S2 Calibration of the imaging sensors.

Fig. S1 Calibration functions for both sensors.

Fig. S2 Four examples of UAS panoramic stitching error.

Fig. S3 ALS plot point clouds and ground level UAS imagery.

Notes S3 Supporting Information references.

Notes S1 Panoramic reprojection to fisheye imagery.

Figure 1 in the main article text shows the image transformation which is detailed mathematically in this note. Because there is not a 1:1 mapping between pixels in the spherical panorama and the reprojected fisheye image, interpolation is required. The interpolation method performed used the *interpolate.griddata* function from the "Scipy" Python package (Virtanen *et al*., 2020), using a 2D linear interpolation. The input panorama was 8,000x4,000 pixels. Post-processing, the final hemispherical image had a size of 4,000x4,000 pixels. Orientation of the final hemispherical photos was obtained by the yaw angle in the metadata of first photo taken, as well as the hemispherical image (Li & Ratti, 2019) as it also saved that metadata.

To transform the equirectangular spherical 2D images into fisheye imagery we first extract the top half. Next the *interpolate.griddata* function is combined with the below expressions to map input pixel locations to corresponding output fisheye locations. The panorama pixel locations correspond to the azimuth or horizontal angle, which varies from 0 to 2π radians. The azimuth angle of a pixel is (Wang, 2019):

Eqn. S1 $$\Theta_i = x_i \cdot \frac{2\pi}{X}$$

Where Θ_i is the azimuth or horizontal angle, in radians, X is the width of the whole equirectangular image and $x_i$is the location of the given pixel in the X-dimension (ranging in this case from 0 to 8,000 pixels). Next, we apply the following expression:

Eqn. S2 $$H(m_i, n_i) \rightarrow \begin{cases} m_i = \frac{Y}{2} + \cos(\Theta_i) \cdot y_i \\ n_i = \frac{Y}{2} - \sin(\Theta_i) \cdot y_i \end{cases}$$

In which $H(m_i, n_i)$ are the new hemispherical (polar) coordinates, Y is the height of the whole equirectangular image and y_i is the position of the pixel in the Y-dimension (i.e., number of rows or radius, ranging in this case from 0 to 2,000 pixels). Note that these are not the usual polar equations as in this case the image starts in the top. In polar coordinates, the origin of the new hemispherical photo is bottom left corner, whereas in our system it was in top left corner. This is the reason for which the minus symbol in the n_i expression.

Notes S2 Calibration of the imaging sensors.

To avoid projection errors, we conducted calibrations on both of our sensors. Firstly, we conducted the calibration following the Hemisfer calibration protocol. For the DHP, this calibration was performed indoors, taking an upward-looking hemispherical photo of 9 markers located in the wall and on the roof of a room, separated by a 10-degree step, ranging from a 0-

degree zenith angle to a 90-degree zenith angle. The relationship between the zenith angle and the radius was obtained in order to obtain the radial distortion (figure S1, a, in blue). A small amount of radial distortion was found, and a custom lens function was defined in Hemisfer for our subsequent analysis.

After trying the same indoor process with the UAS, it was obvious that it was not possible to take a spherical image indoors due to intense drift caused by indoor wind fluxes, turbulences and positioning failure, which resulted in very low stitching quality. Therefore, the process was repeated outdoors, under a bridge. The results showed no radial distortion for the UAS-based hemispherical image after converting the spherical image taken into a digital hemispherical photo (figure S1, a, in orange).

Additionally, we performed another calibration procedure in order to check the previous calibration results. In this case, the analysis was performed using a computational method using the “OpenCV” Python package (Bradski, 2000). The fisheye.calibrate function was used for the DHP sensor and the calibrateCamera function was used for the UAS sensor. With this procedure, we obtained the camera parameters and distortion coefficients for the sensors, information that we then used to obtain the radial distortion of the DHP hemispherical photo (figure S1, b, blue) and the distortion values of the individual images taken by the UAS (not shown).

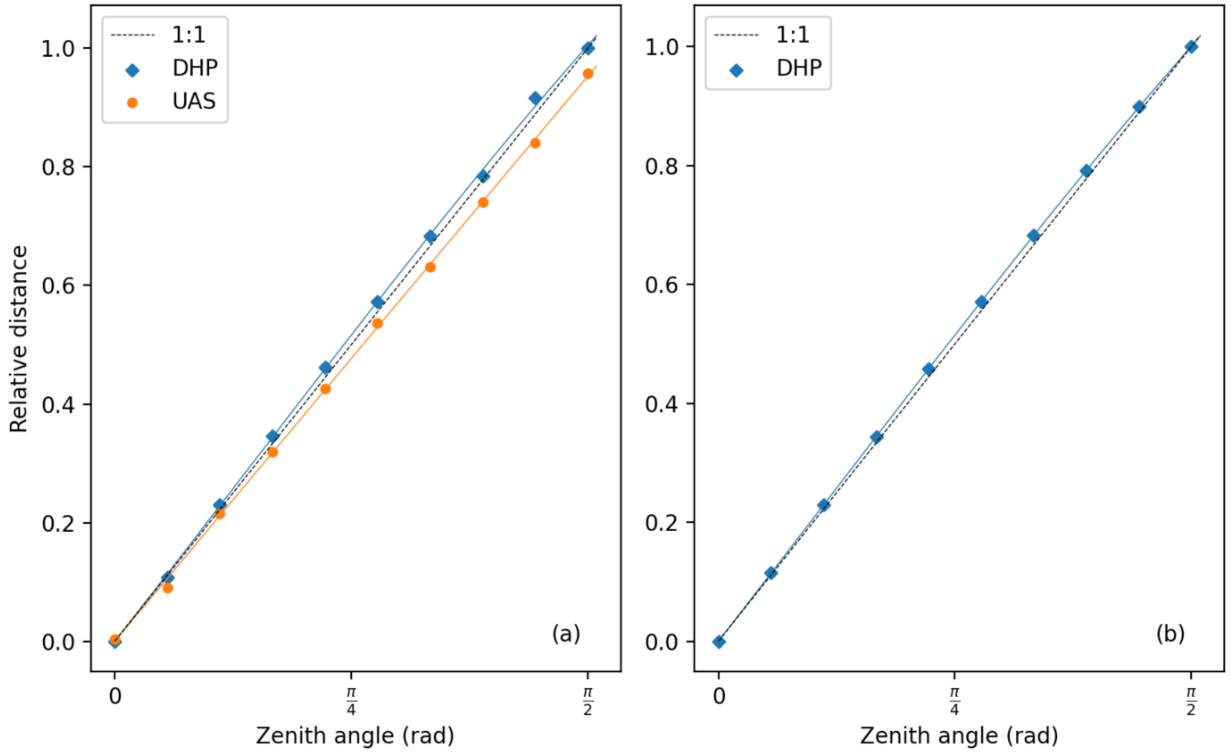

Fig. S1 Calibration functions for both sensors. In blue, digital hemispherical photography (DHP) and in orange, uncrewed aircraft system (UAS). Left panel (a) shows the results of Hemisfer calibration protocol done for both sensors, and right panel (b) shows the results of the computer vision method applied to DHP.

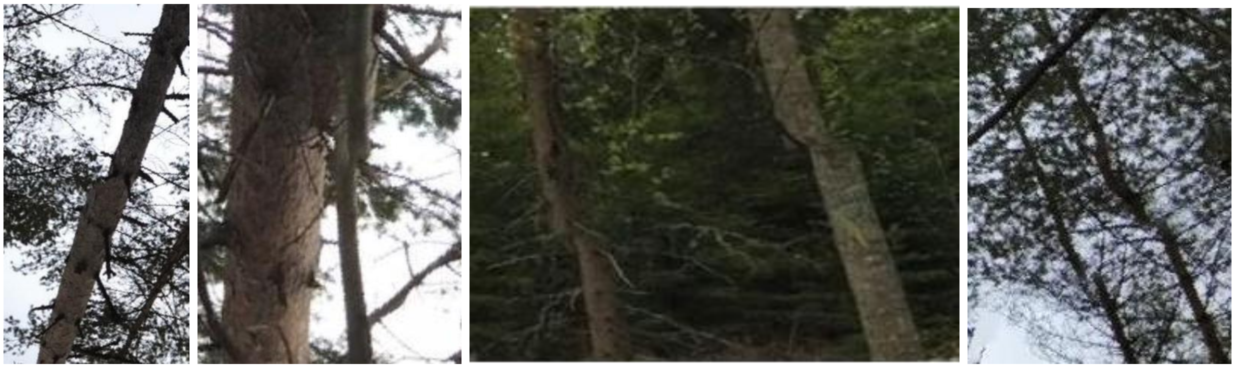

Fig. S2 Four examples of UAS panoramic stitching error.

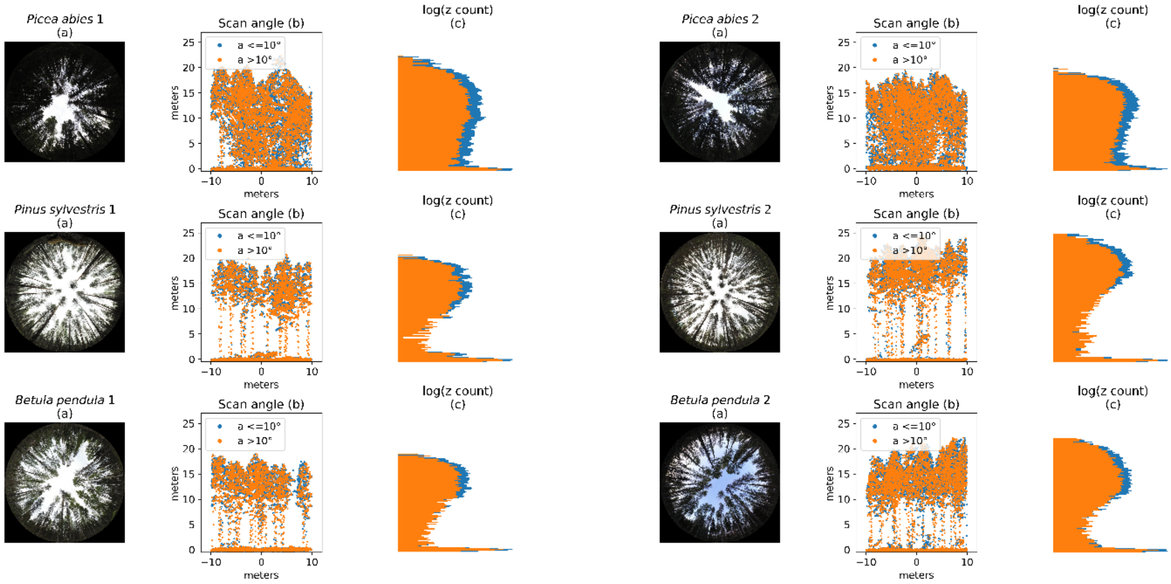

Fig. S3 ALS plot point clouds and ground level UAS imagery. Rows show ground level UAS imagery (a), ALS point clouds (b) and vertical point distributions (c) at each vertical plot location. The point clouds are coloured by a scan angle threshold of 10°, showing a similar distribution of larger and smaller angles for each plot.

Notes S3 Supporting Information references.

Bradski G. 2000. The OpenCV Library. *Dr. Dobb's Journal of Software Tools*.

Li X, Ratti C. 2019. Mapping the spatio-temporal distribution of solar radiation within street canyons of Boston using Google Street View panoramas and building height model. *Landscape and urban planning* **191**: 103387.

Virtanen P, Gommers R, Oliphant TE, Haberland M, Reddy T, Cournapeau D, Burovski E, Peterson P, Weckesser W, Bright J et al. 2020. SciPy 1.0: Fundamental Algorithms for Scientific Computing in Python. *Nature Methods* **17:** 261-272.

Wang H. 2019. Estimating forest attributes from spherical images. Master's Thesis. Faculty of Forestry and Environmental Management, University of New Brunswick, NB, Canada.
